# A Tiered Approach for Assessing Individual and Combined Risk of Pyrethroids Using Human Biomonitoring Data

**DOI:** 10.3390/toxics10080451

**Published:** 2022-08-04

**Authors:** Jose V. Tarazona, Irene Cattaneo, Lars Niemann, Susana Pedraza-Diaz, Maria Carmen González-Caballero, Mercedes de Alba-Gonzalez, Ana Cañas, Noelia Dominguez-Morueco, Marta Esteban-López, Argelia Castaño, Teresa Borges, Andromachi Katsonouri, Konstantinos C. Makris, Ilse Ottenbros, Hans Mol, Annelies De Decker, Bert Morrens, Tamar Berman, Zohar Barnett-Itzhaki, Nicole Probst-Hensch, Samuel Fuhrimann, Janja Snoj Tratnik, Milena Horvat, Loic Rambaud, Margaux Riou, Greet Schoeters, Eva Govarts, Marike Kolossa-Gehring, Till Weber, Petra Apel, Sonia Namorado, Tiina Santonen

**Affiliations:** 1 European Food Safety Authority (EFSA), 43126 Parma, Italy; 2 National Centre for Environmental Health, Instituto de Salud Carlos III, 28220 Madrid, Spain; 3 Department of Safety of Pesticides, German Federal Institute for Risk Assessment, 10589 Berlin, Germany; 4 General-Directorate of Health, Ministry of Health, 1049-005 Lisbon, Portugal; 5 Cyprus State General Laboratory, Ministry of Health, Nicosia 2081, Cyprus; 6 Cyprus International Institute for Environmental and Public Health, Cyprus University of Technology, Limassol 3036, Cyprus; 7 National Institute for Public Health and the Environment (RIVM), Antonie van Leeuwenhoeklaan 9, 3721 Bilthoven, The Netherlands; 8 Wageningen Food Safety Research (WFSR), 6700 Wageningen, The Netherlands; 9 PIH, Knowledge Center for Environment and Health, 2000 Antwerp, Belgium; 10 Department of Sociology, University of Antwerp, 2020 Antwerpen, Belgium; 11 Ministry of Health, Jerusalem 9446724, Israel; 12 Ruppin Research Group in Environmental and Social Sustainability, Ruppin Academic Center, Emek Hefer 4025000, Israel; 13 Swiss Tropical and Public Health Institute, Kreuzstrasse 2, 4123 Allschwil, Switzerland; 14 Jozef Stefan Institute, Department of Environmental Sciences, 1000 Jubljana, Slovenia; 15 Department of Environmental and Occupational Health, Santé Publique France, 12 rue du Val d’Osne, Saint-Maurice, CEDEX, 94415 Paris, France; 16 VITO Health, Flemish Institute for Technological Research (VITO), 2020 Mol, Belgium; 17 German Environment Agency (UBA), 14195 Berlin, Germany; 18 Department of Epidemiology, National Institute of Health Dr. Ricardo Jorge, Avenida Padre Cruz, 1649-016 Lisbon, Portugal; 19 Finnish Institute of Occupational Health, Työterveyslaitos, P.O. Box 40, 00032 Helsinki, Finland

**Keywords:** human biomonitoring, HBM4EU, pyrethroids, guidance values, combined pyrethroid risk assessment, screening assessment, tiered approach, pesticides, biocides, veterinary drugs

## Abstract

Pyrethroids are a major insecticide class, suitable for biomonitoring in humans. Due to similarities in structure and metabolic pathways, urinary metabolites are common to various active substances. A tiered approach is proposed for risk assessment. Tier I was a conservative screening for overall pyrethroid exposure, based on phenoxybenzoic acid metabolites. Subsequently, probabilistic approaches and more specific metabolites were used for refining the risk estimates. Exposure was based on 95th percentiles from HBM4EU aligned studies (2014–2021) covering children in Belgium, Cyprus, France, Israel, Slovenia, and The Netherlands and adults in France, Germany, Israel, and Switzerland. In all children populations, the 95th percentiles for 3-phenoxybenzoic acid (3-PBA) exceeded the screening value. The probabilistic refinement quantified the risk level of the most exposed population (Belgium) at 2% or between 1–0.1% depending on the assumptions. In the substance specific assessments, the 95th percentiles of urinary concentrations in the aligned studies were well below the respective human biomonitoring guidance values (HBM-GVs). Both information sets were combined for refining the combined risk. Overall, the HBM data suggest a low health concern, at population level, related to pyrethroid exposure for the populations covered by the studies, even though a potential risk for highly exposed children cannot be completely excluded. The proposed tiered approach, including a screening step and several refinement options, seems to be a promising tool of scientific and regulatory value in future.

## 1. Introduction

Pyrethroids is a major class of insecticides and are used worldwide in agri- and horticulture, but also for biocidal purposes, including household applications, and as veterinary drugs to control ectoparasites. Pyrethroids are already used as replacement for organophosphate insecticides, and are considered the main alternative to neonicotinoids [1]. Accordingly, their use may be expected to increase in the future, in particular, in those areas such as the European Union (EU) where neonicotinoid use is restricted due to concerns on pollinators [2]. Regarding human biomonitoring, pyrethroids are among the most frequently studied pesticides; this is explained by the widespread use and the public health concern as well as the availability of well-established analytical methods [3]. Variability in exposure levels seems to be high among geographical regions but low between professional users and the general population [4]; and recent studies have confirmed an increase in exposure, at least, in North America [5].

Pyrethroids are synthetic analogues of the pyrethrins originally isolated from the pyrethrum flower (*Chrysanthemum* (also called *Tanacetum*) *cinerariaefolium*). The individual compounds vary in chemical nature and toxic properties [6]. Neurotoxicity is a common feature of pyrethroid toxicity [7,8]. Acute neurotoxicity in humans has been confirmed by case reports [9,10], and also long-term neurotoxic effects have been suggested by epidemiological studies [11], although information on actual exposure levels for these case reports and epidemiological studies is scarce [12]. Evidence on developmental neurotoxicity of pyrethroids comes mainly from experimental systems although there are also some recent epidemiological studies suggesting effects in humans [8]. Other health effects, such as on reproduction, have also been reported and associated to the pyrethroid exposure in epidemiological studies [13,14].

Biomonitoring studies on pyrethroids include screening approaches using 3-phenoxybenzoic acid (3-PBA) [15], analysis based on other metabolites such as 3-(2,2-dichlorovinyl)-2,2-dimethylcyclopropane-1-carboxylic acid (DCCA) [16,17], and approaches based on toxicokinetic models for identifying the contributions from selected pyrethroids [18] or mixtures [19].

Within the framework of HBM4EU (for details see https://www.hbm4eu.eu/; accessed on 25 May 2022), a methodology for setting human biomonitoring guidance values for the general population (HBM-GV_GenPop_) has been proposed [20] and implemented for different priority substances [21,22,23,24], and adapted for applying the Margin of Exposure approach for the pesticide chlorpyrifos [25]. HBM-GV_GenPop_ are epidemiologically and/or toxicologically derived values, mainly related to urine, which can be compared then to internal exposure levels measured in human biomonitoring studies covering the general population. They represent an internal exposure estimate at which the health-based guidance value selected as toxicological reference (in case of dietary exposure to pesticides, the Acceptable Daily Intake—ADI) is not exceeded and, thus, may be considered as safe from a health point of view.

The chemical structure of pyrethroids leads to a large number of isomeric forms, including in some cases enantiomers and cis–trans stereoisomers. Isomers may have significantly different pesticidal activity and toxicity resulting in a variety of marketed active substances, including both individual isomers and racemic mixtures [26]. The ADI for the pyrethroids selected for this assessment ranges between 0.0025 mg/kg bw for lambda-cyhalothrin and 0.05 mg/kg bw for permethrin. These differences in the toxicity of pyrethroids represent a challenge for assessing risk of pyrethroids as a group.

The general molecular structure of pyrethroids is based on two subunits linked by an ester bound. The main subgroup covers structures that are linked to the 3-phenoxybenzyl alcohol moiety, some with cyano or fluoride substitutions, whereas other subgroups include N-hydroxymethyl type, allethrin-type, and tetrafluorobenzyl type pyrethroids [27]. Cleavage of the ester bound is always part of the metabolic pathway [27,28] but not necessarily the first step. Further metabolism of the released alcohol leads to the production and urinary elimination of 3-PBA, in free or conjugated form. This common metabolite is frequently used as marker for pyrethroid exposure [15]. The second moiety may give more specific metabolites although most of them are still common to more than one active substance (see Section 2.2 and Section 3.1 for details).

We are presenting a tiered risk assessment approach, transforming the complexity of pyrethroid uses and metabolism into an opportunity for assessing the combined risk associated to the overall pyrethroid exposure. As the aim is to offer practical solutions that could be implemented in public health programmes, the approach starts with a conservative and easy to implement screening step, complemented with a set of refinement options.

## 2. Materials and Methods

### 2.1. Conceptual Model and Derivation of Provisional HBM Guidance Values

**Figure 1:**
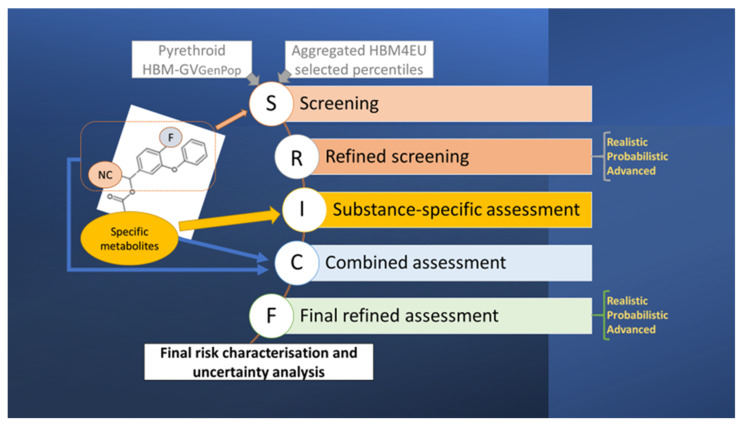
Conceptual model for the proposed tiered biomonitoring pyrethroid risk assessment approach.

Figure 1 summarizes the proposed conceptual approach for the tiered assessment of pyrethroids, compound-specific and combined risk of pyrethroids by comparing measured concentrations of certain metabolites (biomarkers) to HBM-GV. The screening (S) and refined screening (R) phases are based on metabolites linked to the 3-PBA moiety; the substance-specific assessments (I) are based on metabolites from the second moiety. The first step is always based on worst-case assumptions and parameters to establish an HBM screening value for 3-PBA whereas the refinement may rely on more realistic assumptions, probabilistic modelling, or advanced assessment using additional information on use patterns, including measured residues in food. Steps S through I could potentially be sufficient to conclude that there is little concern for the monitored population, so the assessment would be complete. Should this not be the case, the subsequent steps should cover the combination of both sources—C; and the final (F) refinement and uncertainty analysis, which is needed when possible health concerns have been identified in the previous steps.

Within the HBM4EU initiative, three options for the derivation of HBM guidance values in the general population (HBM-GV_GenPop_) have been proposed [20]. Our approach follows the second option, i.e., derivation based on an internationally accepted (external) health-based guidance value as the ADI. This method is similar to the establishment of Biomonitoring Equivalents (BE) adopted by Health Canada [29,30], whereby BE values are also derived for substances without an effect threshold. Briefly, the ADI as external toxicity reference value is translated into a corresponding HBM-GV by means of the following mass balance equation, assuming, as an example, one major metabolite as an exposure biomarker and steady-state conditions (i.e., that a balance exists between the intake of the active substance and the internal concentration/excretion of the metabolite used as a biomarker) [20]:

(1) $$\mathrm{HBM}-\mathrm{GV}\left(\mathrm{GenPop}\right)=\frac{\mathrm{ADI}\;\cdot \left[\frac{\mathrm{MW}\left(\mathrm{Metabolite}\right)\cdot \mathrm{Fue}\left(\mathrm{Metabolite}\right)}{\mathrm{MW}\left(\mathrm{Parent}\;\mathrm{compound}\right)}\right]}{\mathrm{Daily}\;\mathrm{urine}\;\mathrm{volume}\;\mathrm{adjusted}\;\mathrm{to}\;\mathrm{the}\;\mathrm{bw}}$$

The ADI and molecular weights (MW) were extracted from the most recent EFSA or ECHA assessment for each pyrethroid, or from other expert reviews, such as JMPR (Joint FAO/WHO Meeting on Pesticide Residues). The value for the “fraction of urinary excretion” (Fue) was selected from published data covering toxicokinetic studies in humans (see Section 2.2 for details and Section 3.1 for the respective results). For the HBM4EU project, average daily urinary volumes adjusted to bodyweight of 0.03 and 0.02 L/kg bw/d for children and adults, respectively, as proposed by the German HBM Commission have been assumed [20].

Following Equation (1), a screening level was derived for 3-PBA based on conservative approaches, complemented with provisional HBM-GV_GenPop_ established for a number of pyrethroids. In addition, published HBM-GV for deltamethrin and cyfluthrin [24] were used for risk assessment.

Aggregated HBM data (percentiles and their confidence intervals for the full dataset) obtained from HBM4EU-aligned studies were used. These data are reported in Govarts et al. [31]. The 95th percentiles were selected as conservative estimates of population exposure levels for steps S and I. The Risk Characterisation Ratio (RCR) was estimated as the ratio between the 95th percentile and the corresponding screening or HBM-GV_GenPop_ value; RCRs higher than 1 represent potential concerns requiring refinement.

For the probabilistic refinement, full distributions were reconstructed from the percentiles and used for Monte Carlo analysis using the R-program tool developed by EFSA (https://r4eu.efsa.europa.eu/, accessed on 25 May 2022); details for each refinement are provided in the Results section. The same platform was used for probabilistic refinements for other toxicokinetic and toxicodynamic parameters. Advanced refinements were based on complementary information on EU population exposure, specifically on monitored levels in food for the different pyrethroid-active substances.

### 2.2. Data and Information Sources

Pyrethroid metabolites were measured in HBM4EU-aligned studies in adults (age range 20–39 years) and children (4–11 years old) from different European countries, and Israel. These included four studies on adults (DE ESB, FR ESTEBAN, CH HBM4EU study, IL RAV MABAT, ), with an age range of 20–74 years, from France, Germany, Israel, and Switzerland, conducted in the period 2014–2021; and six studies on children (BE 3xG, FR Esteban, CY Organiko, IL RAV MABAT, SI SLO CRP, NL SPECIMEN, ), with an age range of 6–11 years, from Belgium, Cyprus, France, Israel, Slovenia, and The Netherlands, conducted in the period 2014–2020. Table 1 provides summary information on each study and additional details are available in previous HB4EU publications [31,32,33].

Table 2 presents the pyrethroid urinary markers selected in the HBM4EU project and the active substances covered by this risk assessment. In some cases, sum values for Σ(3-PBA + 4-FPBA) and Σ(cis-DCCA + trans-DCCA) were provided. The active substances from the pyrethroid class were selected considering those currently approved as pesticides or biocides in the EU, and with regard to the priorities established under the EU Community control programme on pesticides monitored in food (Commission Implementing Regulation 2020/585). It should be acknowledged that the biomarkers may be also common for other pyrethroids not included in this risk assessment.

The ADIs, and additional information on toxicity and toxicokinetics were retrieved from the European Food Safety Authority (EFSA). Conclusions and related documents published in the EFSA Journal https://efsa.onlinelibrary.wiley.com/journal/18314732 (accessed on 25 May 2022) or publicly available through the Open-EFSA web https://open.efsa.europa.eu/ (accessed on 25 May 2022); and from the European Chemicals Agency (ECHA) assessments on biocides https://echa.europa.eu/es/information-on-chemicals/biocidal-active-substances (accessed on 25 May 2022). References mentioned in these reports, including JMPR and other evaluations, were also considered as needed.

A literature search in the databases Web of Science and SCOPUS was conducted for retrieving toxicokinetic information with the focus on studies in human volunteers.

## 3. Results

### 3.1. Screening and Substance-Specific HBM-GV_GenPop_

Table 3 presents the overview of the proposed biomonitoring guidance values for the various substances. The designation “proposed” indicates that corresponding HBM guidance values were derived as part of HBM4EU work but, unlike the adopted values, have not yet been subjected to a consultation process, they are thus provisional HBM guidance values. If available, specific metabolites are preferred to be used as biomarkers. Data sources for the different parameters and detailed justifications for the selections are provided in the specific subsections below.

#### 3.1.1. Screening Values for 3-PBA Moiety Metabolites

The common metabolite 3-PBA covers a significant number of pyrethroids, which can be extended with the addition of 4-FPBA. Although the toxicological information on 3-PBA is scarce, a recent systematic review [34] has confirmed that this metabolite may contribute to the toxicity of the pyrethroid-active substances but only for some endpoints, such as immunotoxicity, and the effects are only observed at much higher exposure levels than for the parent pyrethroids. This review supports the use of the toxicity reference values established for the parent-active substances for setting guidance values for human biomonitoring. For the different parent compounds that may give this metabolite, the ADI values vary remarkably from 0.0025 mg/kg bw for lambda-cyhalothrin [35] to 0.05 mg/kg bw for permethrin [36]. This is, along with the different molecular weights of the parent substances, one of the two main challenges for application of the urinary mass-balance approach [15,20] in this case. The other is the very different urinary fraction that is excreted as 3-PBA, following oral administration of the various pyrethroid compounds. The respective rates might range from 9% for deltamethrin up to 85% for trans-permethrin [37]. The review by Aylward and co-workers [15] includes data on cypermethrin, deltamethrin, lambda-cyhalothrin, and permethrin. The same data have been used by other authors for developing toxicokinetic profiles. Table 4 includes a summary of previous reviews and original references.

The results confirm large variability among experiments, but also within the same study when the details for each volunteer are presented, in line with previous reviews [18]. For cypermethrin, there are two independent studies, and a factor of 2 between the averaged values is observed. One value is close to the value reported for deltamethrin while the other is almost equivalent to the value reported for lambda-cyhalothrin. The values for permethrin suggest much higher molar rates than for the other pyrethroids, but when data for each individual volunteer are considered, there is an overlap in the ranges observed for permethrin and cypermethrin. Because of these uncertainties, no proper HBM-GV may be established for 3-PBA. Instead, a conservative “screening value” was derived to support Tier I estimates of the overall pyrethroid exposure.

With regard to Equation (1), the use of the lowest ADI (0.0025 mg/kg bw) and the lowest urinary fraction (9%) would be the most conservative assumptions giving the lowest possible “screening values”. This high conservatism is based on the fact that these figures were obtained with different compounds. The resulting urinary screening values of 3-PBA are 3.25 µg/L for children and 4.8 µg/L for adults.

Deterministic refinements can be based on using less conservative assumptions for the urinary fraction. Three options, the 5th percentile, the geomean for averaged values, and the geomean including cis/trans differences were considered. The resulting urinary fractions are 11, 21, and 31%, corresponding to less conservative screening values of 7.6, 14.2, and 22.0 for children and 11.4, 21.3, and 33.0 µg/L for adults, respectively.

**Figure 2:**
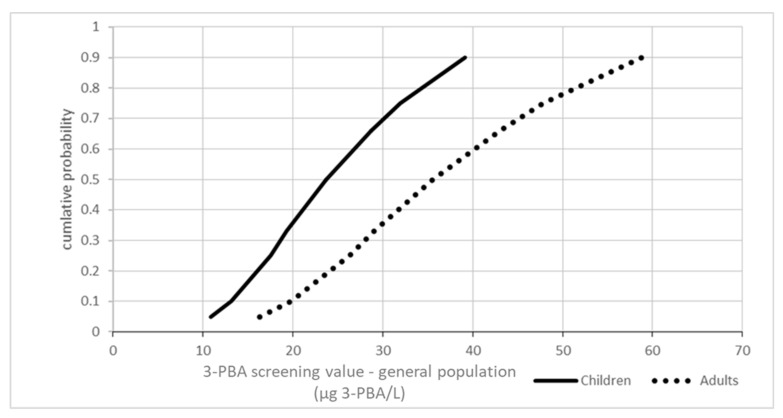
Cumulative probability distribution of the 3-PBA HBM screening value for the general population, accounting for the variability in human toxicokinetics.

A probabilistic approach using Monte Carlo simulation was implemented in the EFSA platform (https://r4eu.efsa.europa.eu/app/montecarlo, accessed on 25 May 2022) with 10,000 iterations and default conditions. Considering the limited number of data, the direct fitting to a distribution was not feasible. Therefore, as a simplified approach, a triangular distribution was used. The selected values for the triangular distribution were the minimum reported value (as surrogate for the 5th percentile), the 50th, and 95th percentiles. The obtained distribution for the 3-PBA screening values is presented in Figure 2.

Theoretically, it should be possible to include also in the probabilistic assessment differences in the ADIs. However, as the differences do not represent variability in the dataset, but real differences in the potency and toxicity of the different active substances, this approach should be used in the risk characterisation phase, not for setting screening HBM-GV_GenPop_ values.

The available information for deriving a screening value for 4-FPBA is limited to a single study with a single volunteer. Considering that this single value is within the range observed for 3-PBA, the worst-case value of 9% was considered as the best alternative for setting Tier I screening values of 16.0 µg 4-FPBA/L for children and of 24.7 µg/L for adults based on the ADI for cyfluthrin of 0.01 mg/kg bw.

#### 3.1.2. Deltamethrin

The following HBM-GVs have been adopted for deltamethrin [24]:

HBM-GV_GenPop_ Adults: 130 µg DBCA/L urine;

HBM-GV_GenPop_ Children: 90 µg DBCA/L urine.

DBCA cis isomer, (cis-3-(2,2-dibromovinyl)-2,2-dimethylcyclopropanoic acid = Br2CAcid) as a substance-specific and sufficiently sensitive metabolite of deltamethrin was used for the HBM-GV derivation. Toxicokinetic studies in humans (five volunteers,3 M + 2 F, age 23–55 a) have shown that after oral exposure to deltamethrin, a mean of 45% of the orally applied dose is excreted as DBCA in urine within 24 h (46 % within 48 h) [37]; thus, the Fue necessary for the calculation according to Equation (1) is 0.45. Neurotoxicity is the critical effect of deltamethrin. In studies with several animal species, acute neurotoxic effects were often observed at doses lower than those leading to other adverse effects. From sub-chronic and chronic studies in dogs, a NOAEL of 1 mg/(kg bw per day) was established including this critical endpoint.

Based on a NOAEL of 1 mg/kg bw per day from various chronic studies with dogs and rats, the Joint Meeting on Pesticide Residues (JMPR) of the World Health Organization (WHO) and the Food and Agriculture Organization of the United Nations (FAO) derived an ADI of 0.01 mg deltamethrin/kg bw, taking into account an overall safety factor of 100 [42]. The approach was followed in deriving an ADI in the context of the EU evaluation of deltamethrin as a plant protection product (draft RAR (renewal assessment report) https://www.efsa.europa.eu/es/consultations/call/180724, accessed on 25 May 2022) with a slightly different selection of studies but leading to an identical ADI.

#### 3.1.3. Cyfluthrin

The following HBM-GVs have been adopted for cyfluthrin [24]:

HBM-GV_GenPop_ Adults: 130 µg 4-FPBA/L urine;

HBM-GV_GenPop_ Children: 80 µg 4-FPBA/L urine.

4-FPBA (4-fluoro-3-phenoxybenzoic acid) is considered as an almost specific biomarker of exposure for cyfluthrin/ß-cyfluthrin in humans [43], as only flumethrin as active ingredient of veterinary drugs is also metabolized to 4-FPBA. Urinary levels of 4-FPBA can be considered to reflect recent exposure to cyfluthrin/beta-cyfluthrin but do not allow for discrimination between exposure to cyfluthrin and beta-cyfluthrin as the 4-FPBA moiety is identical in all cyfluthrin isomers. Limited data from a toxicokinetic study in a single person show that about 25% expressed as a mass fraction of an orally administered cyfluthrin dose or 47% expressed on a molar fraction basis (Fue = 0.47) were recovered in urine within two days [44,45]. It should be noted that considering this uncertainty, a more conservative approach, Fue = 0.09, has been used for setting the screening value of 4-FPBA.

The critical effect of cyfluthrin/ß-cyfluthrin that must be taken into account when deriving a health-based guidance value is neurotoxicity. Quantitatively, beta-cyfluthrin, being the biologically active component of cyfluthrin, is more potent than cyfluthrin with established ADIs of 0.01 mg/kg bw for betacyfluthrin (PPP regulation, https://www.efsa.europa.eu/en/consultations/call/170407, accessed on 25 May 2022) [46] (EC, 2017b; EC, 2020b); and 0.02 mg/kg bw for cyfluthrin (biocides regulation) [47] (EC, 2018b). Since the determination of 4-FPBA in urine does not allow for discrimination between exposure to beta-cyfluthrin or cyfluthrin, respectively, the ADI of 0.01 mg/kg bw for beta-cyfluthrin was used in a conservative approach for setting the HBM-GVs.

The database for cyfluthrin/beta-cyfluthrin concerning toxicity is extensive and the level of confidence with this regard medium to high. Evaluations were performed by several competent authorities within the process of approval procedure for use as biocide or pesticide. However, there is great uncertainty regarding the amount of the cyfluthrin metabolite 4-FPBA excreted in urine by humans, because available information is based on a single study with a single individual only.

#### 3.1.4. Cypermethrin

For cypermethrin, the following provisional HBM-GVs were derived for this risk assessment:

HBM-GV_GenPop_ Adults: 45 µg DCCA/L urine;

HBM-GV_GenPop_ Children: 30 µg DCCA/L urine.

These values are based on the ADI of 0.005 mg/kg bw [48] and 36% recovery of the metabolite DCCA (cis/trans isomers combined) in the urine of human volunteers following a single low oral dose [40,42].

Critical toxic endpoints for the derivation of the ADI were adverse effects on body weight und non-neoplastic kidney findings in a 2-year rat study and neurological signs and lower pup viability in a developmental neurotoxicity study [48].

#### 3.1.5. Lambda-Cyhalothrin

For lambda-cyhalothrin, the following provisional HBM-GVs were derived for this risk assessment:

HBM-GV_GenPop_ Children: 9 µg ClF3CA/L urine;

HBM-GV_GenPop_ Adults: 14 µg ClF3CA/L urine.

These values are based on an ADI of 0.0025 mg/kg bw [35] and 21% recovery of the metabolite CFMP also named ClF3CA as measured during four days in the urine of seven human volunteers following a single low oral dose [49]. This metabolite is common to lambda-cyhalothrin, bifentrin, and tefluthrin.

Brain morphological changes were the critical effects observed in a developmental neurotoxicity study in which the NOAEL was 4.9 mg/kg bw per day. Nevertheless, these effects were covered by the lowest relevant NOAEL of 0.5 mg/kg bw per day as obtained in a multigeneration study in rats with cyhalothrin. At higher dose level, a decrease in body weight gain was noted in offspring. The ADI was derived from this NOAEL, using a higher uncertainty factor than usual. In fact, an additional factor of two was applied to convert from cyhalothrin to the presumably more toxic lambda-cyhalothrin [35].

#### 3.1.6. Permethrin

For permethrin, the following provisional HBM-GVs were derived for this risk assessment:

HBM-GV_GenPop_ Children: 0.32 mg DCCA/L urine;

HBM-GV_GenPop_ Adults: 0.48 mg DCCA/L urine.

These values have been derived on the basis of an ADI of 0.05 mg/kg bw [36] and 36% recovery of the specific metabolite trans/cis DCCA in the urine of human volunteers following a single low oral dose [39,41].

The critical toxicological endpoint for the ADI derivation was neurotoxicity observed in the rat and dog long-term studies (ECHA, 2014 https://echa.europa.eu/es/information-on-chemicals/biocidal-active-substances/-/disas/factsheet/1342/PT18 accessed on 25 May 2022).

#### 3.1.7. Bifenthrin

For bifenthrin, the following provisional HBM-GVs were derived for this risk assessment:

HBM-GV_GenPop_ Children: 60 µg ClF3CA/L urine;

HBM-GV_GenPop_ Adults: 90 µg ClF3CA/L urine.

The ADI value of 0.015 mg/kg bw/day is based on the 1-year dog study with a safety factor of 100, supported by the developmental study in rats [50]. The target effect observed for repeated exposures was tremor indicating neurotoxicity.

It is a challenge to calculate HMB-GVs for bifenthrin. No relevant toxicokinetic human studies to obtain a F_UE_ value for ClF3CA when released from bifenthrin could be identified and retrieved. Therefore, it was not possible to take our usual approach to derive an HBM-GV in this case and, instead, we have used the 21% F_UE_ value as obtained with lambda-cyhalothrin. The latter substance has a similar chemical structure and gives the same metabolite ClF3CA. However, for final calculation, we took the molecular weight and the ADI of bifenthrin into consideration.

#### 3.1.8. Tau-Fluvalinate

For tau-fluvalinate, the following provisional HBM-GVs were derived for this risk assessment:

HBM-GV_GenPop_ Children: conservative GV 0.0064 mg 3-PBA/L, realistic GV 0.022 mg 3-PBA/L;

HBM-GV_GenPop_ Adults: conservative GV 0.0096 mg 3-PBA/L, realistic GV 0.033 mg 3-PBA/L.

This value is based on an ADI of 0.005 mg/kg bw grounded on generic toxicity including a decrease in body weight and food consumption [51] and a recovery of the 3-PBA metabolite (common metabolite for several pyrethroids) of 9% for the most conservative scenario and 31% for the most realistic scenario. There are no human toxicokinetic data on tau-fluvalinate. The toxicokinetic information suggests similar pathways as for other esters containing the phenoxy benzoate moiety, including cypermethrin, tralomethrin, and deltamethrin, supporting the use of the data from other pyrethroids for estimating possible values related to the molar urinary excretion of 3-PBA. Several scenarios and a probability assessment have been conducted to address the uncertainty of these estimations. In the interpretation of the risk characterisation results, it should be considered that 3-PBA is a common metabolite resulting from the exposure to several pyrethroids.

#### 3.1.9. Etofenprox

Lacking human toxicokinetic data on etofenprox and considering the limited toxicokinetic information in animals, the uncertainty in the estimation of the molar urinary excretion of 3-PBA following etofenprox ingestion is too high. Considering that only a part of the radioactivity can be linked to the phenoxy benzoate moiety, that 3-PBA is produced only in some metabolic pathways, and that it may be further metabolised, a worst-case value of 1% could be considered, but with high uncertainty. Considering the large uncertainty and the limited urinary excretion, 3-PBA is not considered a proper marker for this pyrethroid and, therefore, no specific value is proposed, and etofenprox exposure cannot be covered by the human monitoring data.

### 3.2. Screening and Refined Assessments Based on Common Metabolites

In the tier 1 assessment, the 95th percentiles from HBM4EU aligned studies [31] have been compared to the screening values for 3-PBA/4-FPBA. For adults, the highest value of 2.87 µg/L is reported from Israel. Thus, all values were below the screening value and a low concern, with RCR < 1, for adults was identified. A similar situation was observed for the combined screening based on the sum of 3-PBA and 4-FPBA. The maximum reported 95th percentile for adults is 3.13 µg/L reported for Israel, lower than the screening value for 3-PBA that can also be used for the sum of both urinary markers.

The situation is different for children, for which, in general, higher monitoring values were reported, ranging from 3.72 µg/L in Slovenia to 7.05 µg/L in Belgium. The 95th percentiles exceeded the 3-PBA screening value for all children databases (RCRs between 1.14 and 2.17). The highest value was calculated for Belgium, followed by Cyprus (RCRs 1.95). Measured levels for 4-FPBA, in contrast, were well below the proposed screening value for this biomarker. When the screening is conducted for the sum of 3-PBA and 4-FPBA, the RCR are slightly higher, ranging from 1.33 for France and 2.19 for Belgium, with 3-PBA as the most relevant component in all cases.

**Figure 3:**
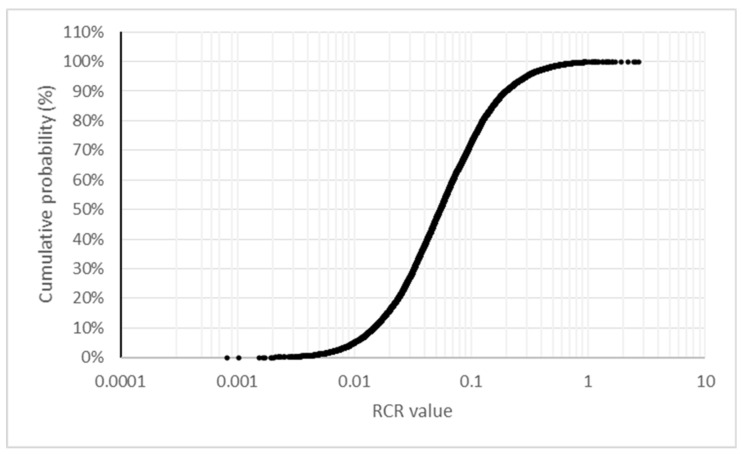
Probabilistic Monte Carlo-based estimation of RCR values for the Belgium children data.

The refinement based on variability in human toxicokinetics presented in Figure 2 suggested a real probability of exceedance below 5%. An additional probabilistic refinement was conducted accounting for the differences in the ADIs between the different pyrethroids in addition to the individual variabilities in the urinary excretion of the marker. As exposure data were aggregated, the first step is to transfer the percentiles into a distribution. The best fit for the Belgian dataset is for a loglogistic distribution (shape 2.025207; scale 1.687245). Individual variability in urinary 3-PBA excretion was modelled taking into account the estimations by Remer et al. [52] and van Haarst et al. [53] as reported by Aylward et al. [15]. The best fit was for a Weibull distribution (shape 2.4009431, scale 0.3465461). Figure 3 summarises the results.

The probabilistic refinement based on variability in human toxicokinetics suggests a potential risk for about 2% of Belgian children if exposure is linked to the more hazardous pyrethroid, lambda-cyhalothrin; the risk of exceedance is reduced to 1–0.1% for assumptions considering a mixed exposure to several pyrethroids with different ADIs.

The levels and variability detected in the HBM4EU studies are similar to those reported in other areas of Europe and elsewhere. For example, the reported 95th percentiles for urinary 3-PBA in children ranged between 0.253 and 18.8 µg/L in Spain [54] and between 1.9 and 20.6 µg/L in the US [55]. The levels in adults are below those reported for US [56], Korea [57], or China [58]. Despite the large number of studies measuring urinary 3-PBA and, at a lesser extent, 4-FPBA concentrations, most of them do not report the 95th percentiles which is the level of exposure selected for our population risk assessment in order to cover the highest exposed group.

### 3.3. Substance-Specific Risk Assessments

A substance-specific risk assessment was conducted for each pyrethroid, comparing the HBM-GV_GenPop_ with the 95th percentiles for the relevant biomarker as obtained in the various HBM4EU aligned studies. The results, expressed as Risk Characterization Ratios (RCR) between the 95th percentile of urine measurements (in µg/L, not adjusted for creatinine) and the HBM-GV_GenPop_ are summarised in Table 5 and described in the sections below.

#### 3.3.1. Deltamethrin

The highest reported 95th percentile values for the populations covered by the aligned studies are 5.37 µg/L of DBCA for adults (France) and 5.32 µg/L of DBCA for children (France). The highest RCRs for deltamethrin were 0.041 for adults and 0.044 for children. It is remarkable that measurements in adults from Germany, Israel, and Switzerland indicated urinary concentrations in adults that were by 80–90% lower than in France. For children, figures from Belgium and The Netherlands, as well as from Cyprus, were comparable and slightly lower than for France, while remarkable low levels were found in Israeli children. Similar results have been reported for other EU countries [59].

As the 95th percentiles of the measured values were well below the HBM-GVs for both, adults and children, there is low concern for deltamethrin exposure in the populations covered by the HBM4EU studies.

#### 3.3.2. Cyfluthrin

The highest reported 95th percentile values for the populations covered by the aligned studies are 0.07 µg/L of 4-FPBA for adults (France) and 0.29 µg/L of 4-FPBA for children (Slovenia); resulting in RCRs well below 1 for these studies. Even though the proposed HBM-GV for cyfluthrin are based on a study in a single human volunteer only, the resulting screening values for 4-FPBA can be used as a conservative approach. As the 95th percentile of the measured values is well below the screening values for both, adults and children, a low concern for cyfluthrin can be confirmed. In fact, most measurements were below detection levels, indicating only sporadic exposure. Similar results have been reported for other EU countries [59] and elsewhere [56,58].

The main uncertainty is related to the toxicokinetics in humans, the value selected for the specific HBM-GV has high uncertainty and, in the opinion of the authors of this study, should be revised as soon as new data or usable PBPK models are available, keeping in mind that a large inter-individual variability among human volunteers has been observed with other pyrethroids. The metabolite is also common to flumethrin, used as a drug in veterinary medicine, and, at least theoretically, part of the urinary levels may correspond to exposure to this substance. Both uncertainties indicate that the assessment is conservative, thus the uncertainty analysis confirms low concern regarding cyfluthrin exposure for the populations covered by the HBM4EU measurements.

#### 3.3.3. Cypermethrin

In the aligned studies in children, the 95th percentiles varied between 3.2 µg/L in France up to 7.5 µg/L in Belgium with values from the Netherlands, Cyprus, and Israel in between. In adults, the highest urinary concentration of DCCA, 3.08 µg/L (95th percentile) was measured, in contrast, in Israel whereas the lowest (0.85 µg/L) was found in Germany.

Based on these data, the highest RCRs for cypermethrin were 0.068 for adults and 0.25 for children. Since all the 95th percentiles of the measured values were below the HBM-GVs for both, adults and children, there is low concern for cypermethrin exposure in the populations covered by the HBM4EU studies even though exposure of children might give a reason for closer monitoring.

It is worth noting that the 95th percentiles from the aligned studies were in good compliance with published data. Couture et al. [60] reported urinary concentrations of up to 8.8 µg/L (median 0.39 µg/L) for trans-DCCA and of up to 3.0 µg/L (median 0.14 µg/L) for cis-DCCA for children and adults from a rural area in Quebec. In Brittany in Western France, urine samples were collected from six-year-old children between 2009 and 2012, rather low 95th percentiles for cis-DCCA (0.49 µg/L) and trans-DCCA (1.75 µg/L) were established [61].

In contrast, on the island of Taiwan, Simaremare and co-workers [15] detected very high mean urinary concentrations of 2.71 µg/L for cis-DCCA and of 19.25 µg/L for trans-DCCA in a cohort of 30 pregnant women (95th percentiles not given). Maximum concentrations of 34.6 µg/L for cis-DCCA and even of 71.3 µg/L for trans-DCCA would have exceeded the proposed HBM-GV and might indicate a health concern.

#### 3.3.4. Lambda-Cyhalothrin

The highest reported 95th percentile values of Cl3FCA for the populations covered by the HBM4EU Aligned Studies were 1.05 µg/L of Cl3FCA for adults as found in a study in Israel and 1.28 µg/L for children in a study from The Netherlands.

The highest RCRs for lambda-cyhalothrin were 0.075 for adults and 0.142 for children.

The proposed HBM-GV_Gen Pop_ for lambda-cyhalothrin are based on measured urinary levels of the specific metabolite Cl3FCA in studies with human volunteers. This metabolite is also common with bifenthrin and tefluthrin which are, however, much less frequently used than lambda–cyhalothrin; and also, less frequently found at relevant residue levels in food [62].

The results indicate low concern for lambda-cyhalothrin exposure in the populations included in the HBM4EU-aligned studies.

#### 3.3.5. Permethrin

The highest reported 95th percentile values for the populations covered by this HBM4EU assessment are 3.08 µg/L of DCCA for adults (obtained in Israel) and 7.52 µg/L of DCCA for children (obtained in Belgium). For adults, the figures are higher in Israel than in France, Switzerland, and Germany while for children, figures are higher in Belgium when compared to The Netherlands, Cyprus, France, and Israel.

The highest RCRs for permethrin were 0.006 for adults and 0.02 for children which demonstrates that there is no health concern for both population groups covered in the HBM4EU-aligned studies. It should be noted that DCCA levels may be also the result of cypermethrin exposure.

#### 3.3.6. Bifenthrin

The Cl3FCA reported under 3.2.4 were also compared with the proposed HBM-GV_GenPop_ value for bifenthrin.

The highest RCRs for bifenthrin were 0.012 for adults and 0.021 for children, indicating low concern for bifenthrin exposure in the populations included in the HBM4EU Aligned Studies. It should be highlighted that exposure to bifenthrin is practically negligible in the EU [62]; and urinary levels of Cl3FCA may result from exposure to lambda-cyhalothrin or perhaps tefluthrin.

#### 3.3.7. Tau-Fluvalinate

In addition to its use in plant protection products, tau-fluvalinate is also applied as a miticide in apiculture. It is frequently detected in beeswax [63], and honey with frequent exceedances of maximum residue limits [64]. Consumer exposure is mostly associated to residues in fruits (e.g., pears, oranges) with low detection frequencies and no risk concerns in the EU [64].

There is no specific metabolite for tau-fluvalinate that could be used as biomarker. Therefore, risk assessment of tau-fluvalinate is based on determination of 3-PBA, but using the ADI for this pyrethroid in the calculation. The 95th percentiles for all monitored groups provided by HBM4EU are well below the proposed realistic HBM-GV_GenPop_; however, in the specific case of the Belgium children database, the 95th percentile, 7.05 µg 3-PBA/l, is slightly higher than the proposed conservative GV for children, 6.4 µg 3-PBA/l. When the upper limit of the 95th percentile is considered, accounting for the exposure uncertainty, the realistic GV is exceeded for the Israel database and the conservative GV is exceeded for all children databases except Slovenia.

The biomarker for tau-fluvalinate is based on a common metabolite for several pyrethroids. In the case of the maximum 95th percentile (Belgium children), the exceedance of the tau-fluvalinate conservative GV would require that over 90% of the measured biomarker results from tau-fluvalinate exposure; and a contribution higher than 26% would be required for exceeding the maximum upper confident limit of the 95th percentile (Israel children). This is unlikely considering that the recent EU reports on pesticides residues in food [62,64] indicate consumer exposure levels below 0.006 µg tau-fluvalinate per kg body weight and day.

Based on the above considerations, it is concluded that exposure to tau-fluvalinate, in isolation, does not pose a risk for the populations covered by the HBM4EU project and included in this assessment.

### 3.4. Combined Risk Assessment

As indicated in Table 2, the available urine biomarkers for pyrethroid exposure are mostly common metabolites and cannot be unequivocally linked to the exposure to a specific parent pyrethroid. However, in combination, they can offer information on the level of exposure to the most toxic active substances. As a risk for cyfluthrin has been already excluded, the refinement was focused on those covered by 3-PBA. In a first step, CIF3CA, DCCA, and DBCA levels were considered for estimating the maximum possible contribution of λ-cyhalothrin, cypermethrin, and deltamethrin, respectively, using the following equation:

(2) $$\mathrm{Contribution}\;P_{i}\;\mathrm{to}\;3\mathrm{PBA}\;\mathrm{levels}=\frac{3\mathrm{PBA}\;\mathrm{based}\;P_{i}\;\mathrm{HBM}-\mathrm{GV}}{\begin{array}{c} \mathrm{RCR}\;P_{i}\;\mathrm{based}\;\mathrm{on}\;\mathrm{selective}\;\mathrm{metabolite}\; \end{array}}$$

where, P_i_ represents the selected pyrethroid “i”; the 3-PBA-based HBM-GV were estimated using the 9% urinary fraction for 3-PBA and the ADI for P_i_. It should be noted that as the ADI for each pyrethroid is used for this estimation, the 3-PBA-based HBM-GV is similar to the 3-PBA screening HBM only in the case of lambda-cyhalothrin.

Then, the screening RCR value for 3-PBA was refined considering each pyrethroid contribution and relative potency in comparison to lambda-cyhalothrin, based on the ADI ratio:

Refined RCR = Screening RCR × (∑ (Contribution P_i_ to 3-PBA levels/3-PBA level) × P_i_ relative potency) (3)

This first step already confirmed that RCR values were below 1 for all children 95th percentiles except in Belgium where a refined RCR value of 1.11 was calculated. In a further refinement, relative potencies within the pyrethroids covered by the same selective biomarker were considered. These complementary estimations concluded that the RCR for the Belgium children population will be lower than 1 if realistic assumptions are considered, for example, if the contribution of tau-fluvalinate to the 3-PBA levels is below 65%, as supported by food monitoring results, or if the contribution of permethrin to DCCA levels is above 50%.

### 3.5. Overall Discussion, Uncertainty Assessment, and Final Risk Characterisation

The HBM4EU-aligned studies included in this risk assessment represent regional and national population groups within the EU, in Switzerland, and Israel. The EU population is covered by the same rules and authorisation lists for pesticide and biocide active substances, a single market, common maximum pesticide residue levels, and free transfer of goods; while the non-EU countries have their own internal rules. Nevertheless, no clear differences in pyrethroid-related risk are observed between the EU and the included non-EU populations. As expected, exposure may be different in other jurisdictions, and, in fact, other studies have reported exposure levels in America and Asia above those from HBM4EU-aligned studies [56,57,58]. A more common finding is that exposure levels and the risk for children are higher than those for adults, suggesting to perform further studies in this sensitive population group.

Human biomonitoring data reflect the aggregated exposure from all exposure routes. For pyrethroids, dietary exposure is expected to be the main exposure route for the general population, complemented by environmental, including indoor, exposure [55]. In addition, occupational (dermal and inhalation) exposure is relevant for specific groups, from farmers to those wearing treated clothing (e.g., military, forest workers) or those regularly working in treated closed environments such as flight attendants [65]. Our risk assessment for the general population assumes dietary exposure as the relevant route; this assumption is supported by the comparison of the central tendency and the spread of the aggregated data distributions through the ratios between 95th/50th and 50th/5th percentiles. These ratios are similar for adults and children and mostly between 4 and 6 for all pyrethroid biomarkers and studies. The values are compatible with the combination of differences in levels in food, dietary habits, and intraspecies variability.

While the results of the compound-specific assessments suggest low exposure and the absence of health risks, the screening assessment, based on the common marker, 3-PBA, identified possible concerns for children. Different factors could explain this difference. The first and most evident is that 3-PBA represents combined exposure to several pyrethroids, including others not evaluated in this study. In addition, there are differences in the assumptions and level of conservativeness. In order to address both hypotheses, we compared the RCRs for individual pyrethroids based on the more specific metabolites with the RCR based on 3-PBA. The focus was on children and sufficient information was available for Israel, The Netherlands, Belgium, and Cyprus. The lambda- cyhalothrin RCR was used for ClF3CA, and the cypermethrin RCR was used for DCCA. The sum of RCRs for children were between 0.27 and 0.37, indicating low concern and clearly below the RCRs for 3-PBA screening which were based on the conservative value of 9% urinary excretion. However, when a more realistic 3-PBA urinary excretion ratio of 21% was used, the 3-PBA RCRs became closer, ranging between 0.33 and 0.49, suggesting that our selection has in fact covered the most relevant pyrethroids regarding dietary exposure.

The allocation of a suitable human biomarker to a pyrethroid-active substance represents a main element of uncertainty for pyrethroid risk assessments based on human biomonitoring. The general molecular structure of pyrethroids is based on two subunits linked by an ester bound. The main subgroup covers structures that are linked to the 3-phenoxybenzyl alcohol moiety, some with α-cyano or fluoride substitutions, whereas other subgroups include N-hydroxymethyl type, allethrin-type, and tetrafluorobenzyl-type pyrethroids [27]. The metabolic pathways have been extensively studied and reviewed [27,28]. Cleavage of the ester bound is always part of the metabolic pathway but not necessarily the first step. 3-PBA can be further hydrolysed to 4′-OH-3-PBA. Some pyrethroids (e.g., cypermethrin and deltamethrin) contain an α-cyano group in the 3-phenoxybenzyl moiety that undergoes subsequent hydrolysis, generating 3-PBA. However, the metabolic pathway is very complex, and some pathways include modifications in the phenoxy benzoate moiety prior to the cleavage of the ester bound, with no formation of 3-PBA or its -OH form. Sometimes, 4′-OH-3-PBA can be formed directly, without previous hydrolysis of 3-PBA. The commonalities and complexities described above provide mechanistic support to our conceptual approach for combining Fue values from different pyrethroids in the screening phase and the probabilistic refinement. In the case of etofenprox, the 3-phenoxybenzyl alcohol moiety is linked to the second moiety through an ether bound, instead of an ester group; in this case, the formation of 3-PBA requires the oxidation of the benzylic carbon, followed by hydrolysis of the resultant ester [66]; this metabolic difference supports our conclusion that 3-PBA is not an appropriate biomarker for etofenprox.

The common metabolite 3-PBA has been frequently used as marker for pyrethroid exposure [15,67]. Some pyrethroids in this subgroup (cyfluthrin, flumethrin) contain a 4-fluoro substitution, which is not subject to hydrolysis, leading to a complementary biomarker. Our conceptual model includes 4-FPBA in the screening phase, in order to cover also these fluoro-substituted pyrethroids.

The second moiety may give more specific metabolites, although most of them are still common to more than one active substance. The conceptual model with screening and several refinement options, combining screening and compound-specific markers, offers a fit for purpose approach, covering the combined risk of dietary pyrethroid exposure at population level. It should be acknowledged that the biomarkers also cover pyrethroids not included in this risk assessment. For example, 4-FPBA is also a metabolite of flumethrin that is authorised in the EU as a drug in veterinary medicine; DCCA is a metabolite also released from transfluthrin (cis isomer) and allethrin, phenothrin, pyrethrum, resmethrin, and tetramethrin (trans isomer); and 3-PBA is a common metabolite for over a dozen commercialised pyrethroids [27,28]. Exposure to these pesticides due to other uses, such as in veterinary medicine, cannot be fully excluded but the results above suggest that their contributions to the overall burden for most consumers is low; and in any case, the proposed methodological approach will cover also the risk for unselected pyrethroids, if relevant.

The RCRs presented in this study are based on the 95th percentiles, excluding, by definition, 5% of the individuals within each population group which might be at risk in case of an RCR equal to 1. The RCRs based on the 50th percentiles would be on average 5 times lower, providing an additional margin for the averaged population risk. As illustrated in Figure 3, the proposed conceptual model suggests the use of probabilistic estimations for quantifying the actual population risk. The 95% confidence intervals for the 95th percentiles were very wide for 3-PBA and DCCA, particularly in the case of Israel, while other studies have reported individual levels which were one order of magnitude above the 95th percentile [68]. These high levels may be the result of dietary ingestion combined with additional dermal or inhalation exposures from, e.g., parallel occupational use. Some uses, such as for pet treatment or in-house biocidal applications, may result in short-term exposures while occupational and environmental exposure may result in both short-term or long-term exposure depending on the use patterns. The urinary elimination of pyrethroid metabolites is relatively fast, and as the biomonitoring information is based on single samples per individual, it is not possible to conclude if the 5% subgroup not covered by the RCRs correspond to individuals with constant high exposures or to sporadic exposures around the sampling time.

Another source of uncertainty is the limited information on toxicokinetic in humans, as well as the reported high individual variability. As shown in Figure 2, the proposed conceptual model offers a probabilistic refinement for covering this uncertainty. It should be mentioned that all studies on human volunteers were conducted in adults, thus the uncertainty is particularly high for children. The proposed urinary excretion factors are conservative. In fact, the population group with highest metabolite levels is expected to include individuals with high exposure levels as well as those with the highest urinary metabolite fractions. The reported RCR would be overly conservative for the second group. In addition, for the evaluation of human biomonitoring results, it must be considered that considerable variation in the results for individual spot urine must be expected. Our assessment assumes steady state conditions; not considering variability regarding to the sampling day, sample size, or variability in the levels of pyrethroid residues in the diet.

Finally, there is an intrinsic methodological source of uncertainty linked to the selection of ADIs for animal studies as toxicological reference values; in addition, all pyrethroids have not been yet tested for developmental neurotoxicity which may result in lower ADIs than ADIs which are based on adult neurotoxicity. There is a bulk of published epidemiological studies, as well as endpoint specific reviews [14] and systematic reviews [69], reporting associations between current levels of pyrethroid exposure, assessed through human biomarkers, and health effects. Conducting a review of all these claims is outside the scope of this study; EFSA is currently assessing the information on 3-PBA and other metabolites [34] and the available epidemiological studies are considered in EU evaluations, which are updated on regular basis. In order to support public health considerations, we used the most recently established ADI for the respective substance for setting the provisional HBM-GV_GenPop_. The proposed conceptual model and methodological approach can be easily adapted by taking on board new toxicological and toxicokinetic information.

The annual post-marketing risk assessments based on monitoring residues in food also indicate low concerns regarding the exposure of the EU population to pyrethroids [62,64] and, thus, are in line with the results obtained by means of HBM. However, the results of monitoring are presented for each pyrethroid-active substance alone, and the overall risk of combined pyrethroid exposure is not addressed.

## 4. Conclusions

3-phenoxybenzoic acid (3-PBA) is a common metabolite of most pyrethroids, e.g., of (lambda)-cyhalothrin, cypermethrin, deltamethrin, and permethrin which are all of particular interest under HBM4EU and are expected to contribute most to the total intake of pyrethroids by the European population. For Tier 1 assessment of overall pyrethroid exposure, a very conservative screening level for 3-PBA was established. On this basis, the proposed conceptual approach identified a potential concern for all children studies as the 95th percentiles exceeded the screening value of 3.25 µg 3-PBA/L urine. The probabilistic refinement quantified the risk level of the most exposed population (Belgium) at about 2%, assuming that exposure is only to the most toxic pyrethroid; reduced to 1–0.1%, in case of exposure to a mixture of pyrethroids.

The screening level could be refined and complemented in a tiered approach, incorporating other, less common and more substance-specific metabolites. No concerns were identified in these Tier II assessments; and the integration of RCRs based on 3-PBA with the sum of RCRs based on the more specific metabolites was sufficient to conclude that there is low concern also for the combined exposure to pyrethroids. An uncertainty analysis has been performed revealing some sources of doubt but, in principle, did not put the overall results into question.

It is worth noting that, although no exceedances of the risk levels have been observed in the HBM4EU aligned studies, the combined risk is below but close to the acceptability threshold, particularly for children. As other studies have reported higher values, both in the EU and abroad, monitoring campaigns should be maintained and extended to other areas in more countries to ensure proper consumer protection.

## Figures and Tables

**Table 1:** Summary description of the characteristics of the HBM4EU-aligned studies used for this risk assessment. More information on the cohorts can be found in Govarts et al. [31].

Study Acronym	Location	Geographical Coverage	Region	Study Design	Sampling Period	Age Range
**Children**						
3xG	Belgium	Regional	Dessel, Mol, Retie	Longitudinal	01/2019–06/2021	6–8
ESTEBAN	France	National	Mainland	Cross-sectional	04/2014–03/2016	6–11
ORGANIKO	Cyprus	Regional	Limassol	Cross-over	01/2017–04/2017	10–11
RAV MABAT	Israel	National	-	Cross-sectional	2015–2016	4–11
SLO CRP	Slovenia	Regional	Mura region	Cross-sectional	01/2018–06/2018	7–10
SPECIMEn-NL	The Netherlands	Regional	Central-East	Cross-sectional	01/2020–03/2020	6–11
**Adults**						
ESB	Germany	Regional	Münster	Cross-sectional	Earliest samples from 1981, ongoing study	20–29
ESTEBAN	France	National	Mainland France	Cross-sectional	04/2014–03/2016	18–74
HBM4EU-study for Switzerland	Switzerland	Regional	Basel	Cross-sectional	01/2020–10/2020	20–39
RAV MABAT	Israel	National	-	Cross-sectional	2015–2016	20–39

**Table 2:** Selected pyrethroid urinary markers and active substances.

Biomarker	Name	Parent Pyrethroids
3-PBA	3-phenoxybenzoic acid	Many, e.g., cypermethrin, deltamethrin, etofenprox, fenpropathrin, fenvalerate, esfenvalerate, lambda-cyhalothrin, permethrin, tau-fluvalinate
4-FPBA	4-fluoro-3-phenoxybenzoic acid	cyfluthrin
CIF3CA *	cis-3-[2-chloro-3,3,3-trifluoroprop-1-enyl]-2,2-dimethylcyclopropanecarboxylic acid	bifenthrin, lambda-cyhalothrin and tefluthrin
DBCA (cis isomer)	cis-3-(2,2-dibromovinyl)-2,2-dimethylcyclopropane-1-carboxylic acid	deltamethrin
DCCA (sum of cis and trans)	3-(2,2-dichlorovinyl)-2,2-dimethylcyclopropane-1-carboxylic acid	cyfluthrin, cypermethrin, and permethrin

* ClF3CA can also be found with the acronym CFMP.

**Table 3:** Biomarkers for pyrethroid exposure, HBM-GVs, and their basis.

Active Substance	ADI mg/kg bw Source *	Biomarker	Fue * %	Proposed HBM-GV_GenPop_ µg Metabolite/L Urine	Comments
Deltamethrin	0.01 HBM4EU	DBCA	45	90 (children) ^†^ 130 (adults) ^†^	Specific metabolite #
Cyfluthrin	0.01 HBM4EU	4-FPBA	47	80 (children) ^†^ 130 (adults) ^†^	Screening and specific metabolite #
Cypermethrin	0.005 EFSA	DCCA	36	30 (children) 45 (adults)	Sum of cis/trans-DCCA
Lambda-cyhalothrin	0.0025 EFSA	CIF3CA	21	9 (children) 14 (adults)	
Permethrin	0.05 ECHA	DCCA	36	320 (children) 480 (adults)	Sum of cis/trans-DCCA
Bifenthrin	0.015 EFSA	CIF3CA	21	60 (children) 90 (adults)	Fue inferred from lambda-cyhalothrin
Tau-fluvalinate	0.005 EFSA	3-PBA	9–31	6.4–22 (children) 9.6–33 (adults)	Screening and specific metabolite #
Etofenprox	0.03 EFSA	3-PBA	1	low reliability	Excluded from this risk assessment

* References and additional details on the ADI source and on the fraction of urinary excretion (Fue) values are provided in the text under Section 3.1 for each pyrethroid-active substance. # Specific metabolite for the selected pyrethroids but may be common with pyrethroids not included in the list. ^†^ Values adopted within HBM4EU.

**Table 4:** Extraction of data on molar 3-PBA urinary excretion rates (moles of 3-PBA in urine per mole of orally administered parent compound).

	Aylward et al. [15]		Côté et al. [38]		Quindroit et al. [19]		Côté and Bouchard [18]	
	Reported Value	Original Reference	Reported Value	Original Reference	Reported Value	Original Reference	Reported Value	Original Reference
Cypermethrin	0.13 0.27	[39,40]	0.129	[39]	trans 0.39 cis 0.16	[39,40]	0.05–0.55	[40]
Deltamethrin	0.09	[37]			0.15	[37]		
Lambda-cyhalothrin	0.251	Marsh et al. 1994 ^#^						
Permethrin	0.457	[41]	0.129 *	[39]	trans 0.85 cis 0.37	[37]	0.32–0.78	[41]

^#^ Unpublished. * From cypermethrin.

**Table 5:** Risk characterization ratios (RCR) for substance specific biomarkers. NR indicates that the 95th percentile could not be estimated due to low detection frequency.

Biomarker	ClF3CA		DBCA	DCCA		4-FPBA
Active Substance	Lambda-Cyhalothrin	Bifenthrin	Deltamethrin	Cypermethrin	Permethrin	Cyfluthrin
Children (age 6 to 11 years)						
Israel	0.085	0.013	0.011	0.17	0.016	0.013
Netherlands	0.142	0.021	0.042	0.15	0.014	NR
Belgium	0.086	0.013	0.034	0.25	0.02	NR
Cyprus	0.029	0.004	0.044	0.20	0.02	NR
France	-	-	0.059	0.108	0.01	0.004
Slovenia	NR	NR	NR	NR	NR	0.018
Adults						
Switzerland	0.031	0.005	0.0068	0.036	0.003	NR
Germany	0.019	0.003	0.0041	0.019	0.003	NR
Israel	0.075	0.012	0.0032	0.068	0.006	NR
France	-	-	0.041	0.053	0.005	0.003

## Data Availability

Summary of EFSA toxicity assessments and monitored levels in food are available through the documents published in the EFSA Journal https://efsa.onlinelibrary.wiley.com/journal/18314732 (accessed on 25 May 2022) at Open-EFSA web https://open.efsa.europa.eu/ (accessed on 25 May 2022), or Zenodo https://zenodo.org/record/6322020#.YkSAbChBzD4 (accessed on 25 May 2022).
